# A radiotherapy staff experience of gratitude during COVID-19 pandemic

**DOI:** 10.1016/j.tipsro.2021.04.002

**Published:** 2021-05-04

**Authors:** Elisa Marconi, Silvia Chiesa, Loredana Dinapoli, Elisabetta Lepre, Luca Tagliaferri, Mario Balducci, Vincenzo Frascino, Calogero Casà, Daniela Pia Rosaria Chieffo, Maria Antonietta Gambacorta, Vincenzo Valentini

**Affiliations:** a UOC di Radioterapia Oncologica, Dipartimento Diagnostica per Immagini, Radioterapia Oncologica ed Ematologia, Fondazione Policlinico Universitario Agostino Gemelli IRCCS, Roma, Italy; b UOS di Psicologia Clinica, Fondazione Policlinico Universitario Agostino Gemelli IRCCS, Roma, Italy; c Università Cattolica del Sacro Cuore, Roma, Italy

**Keywords:** Radiotherapy, Gratitude, COVID-19, Pandemic, Healthcare staff, Teamwork, Burn-out, Psychological distress, Group contact, Technology

## Abstract

• Medical staff from oncology units are at high risk of burnout during COVID-19 pandemic.
• The sense of belonging to the group and the contacting emotions play a central role in reducing these risks.
• Gratitude is related to well-involving people who practice and receive it.
• During lockdown, we adopted gratitude-focused “inter-group contact” tool.

The impact of the COVID-19 pandemic on cancer patients can be high in terms of anxiety, fear and psychological distress [1]. Medical staff from frontline wards, especially oncology units, are at high risk of infection and burnout [2]. Psychological needs grow both for cancer patients and for health-care workers [3].

In some cancer services, the risk of virus infection has led to changes in habits and care plans [4], resulting in disorientation for healthcare staff. In Radiotherapy services the daily interchange of patients has continually exposed health-care workers to frightened, and potentially infected people. This ambivalent aspect between the need for reassurance and personal fear is a psychological aspect that has not yet been explored in the literature [5].

In Italy, the health system emergency has required timely interventions [6] aimed to optimize assistance [7]. A reduction in contact between colleagues can negatively affect teamwork, which is crucial in multidisciplinary teams such as Radiotherapy [8].

Literature in psychosocial sciences has shown that stress [9], fear [10], or emergency [11] influence human relationships; research shows that the sense of belonging to the group and the contact with others’ emotions [12] play a central role in reducing these risks [13]. Studies suggest that workplaces aiming to increase job satisfaction can do so, through well-organized gratitude interventions [14]. Gratitude is also related to well-being, that involves both the people who practise and receive it [15]. It is useful for healthcare professionals to relieve the fatigue of daily commitment and restore meaning to their work [16]*.*

Therefore, during lockdown, it was decided to use a gratitude-focused “inter-group contact” tool [17] to increase group identity and mutual trust, rediscovering the pleasure of being part of a team.

The project was conducted at the Agostino Gemelli IRCCS University Hospital in the Gemelli A.R.T. - Advanced Radiation Therapy- from 1 April 2020, in the midst of the COVID-19 emergency, to today.

This project consisted in the creation of a column, a WhatsApp broadcast, in which a daily message mainly in JPEG format was published: cards composed by letters, emails, images, music, or videos accompanied by a short reflection. The messages of gratitude, written or sent by radiotherapy patients over the years or during the pandemic, were collected by a dedicated team and with the support of all the operators involved (doctors, nurses, technicians, psychologists, administrative staff). Gratitude-oriented messages can help workers find gratification and rediscover meaning in their work (Fig. 1).

**Fig. 1:**
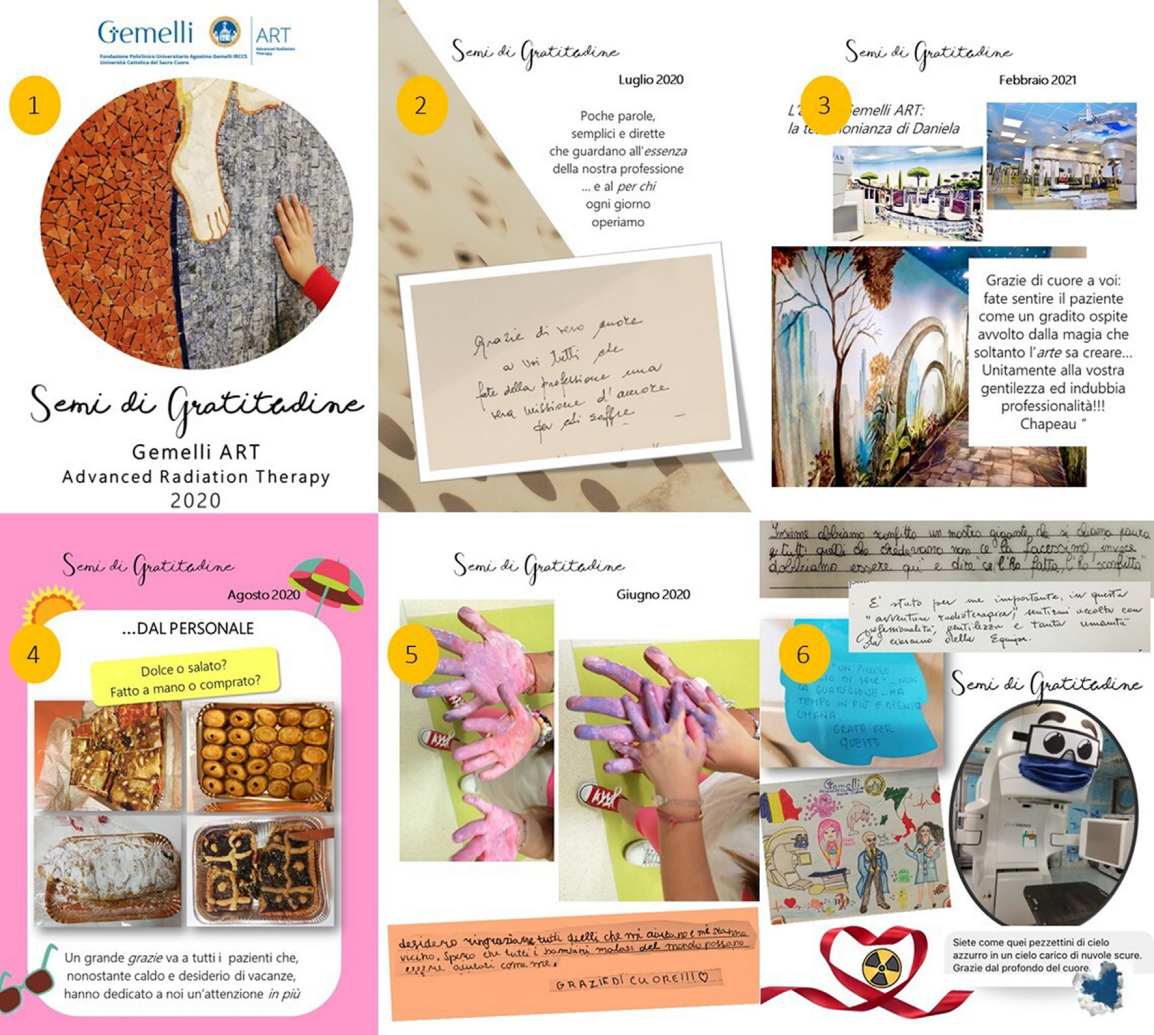
1. Seeds of Gratitude column cover; 2. “Heartfelt thanks to all of you who make your profession a mission of love”; 3. “Many thanks to you: you make the patient feel like a welcome guest surrounded by the magic that only art can create… Together with your kindness and unquestionable professionalism!!! Chapeau”; 4. From the health staff: “A big thank you goes out to all the patients who, despite the heat and desire for vacations, have given us extra attention”; 5. From a pediatric patient: “I would like to thank everyone who helps me and stands by me. I hope that all the sick children in the world can be helped like me. THANK YOU FROM THE BOTTOM OF MY HEART!!!”; 6. Some images from various cards.

In May 2020, when phase two started in Italy, the participants were surveyed on the satisfaction of the project: 87.9% rated the experience satisfaction as ≥ 7, 89.6% wanted to continue the experience, which is currently ongoing with one message per week.

“Seeds of Gratitude” is oriented to include patients’ feelings and to “irrigate” the sense of belonging and group unity, to support healthcare personnel by using affordable technology, and it is now an online book [18]. This experience could be easily extended to other centers that wish to improve teamwork, particularly during periods of uncertainty and high stress.

## Declaration of Competing Interest

The authors declare that they have no known competing financial interests or personal relationships that could have appeared to influence the work reported in this paper.

## References

[1] Tsamakis K., Gavriatopoulou M., Schizas D., Stravodimou A., Mougkou A., Tsiptsios D. (2020). Oncology during the COVID-19 pandemic: Challenges, dilemmas and the psychosocial impact on cancer patients (Review). Oncol Lett.

[2] Wu Y., Wang J., Luo C., Hu S., Lin X.i., Anderson A.E. (2020). A Comparison of Burnout Frequency Among Oncology Physicians and Nurses Working on the Frontline and Usual Wards During the COVID-19 Epidemic in Wuhan, China. J Pain Symptom Manage.

[3] Jones D., Neal R.D., Duffy S.R.G., Scott S.E., Whitaker K.L., Brain K. (2020). Impact of the COVID-19 pandemic on the symptomatic diagnosis of cancer: the view from primary care. Lancet Oncol.

[4] Leung M.S.T., Lin S.G., Chow J., Harky A. (2020). COVID-19 and Oncology: Service transformation during pandemic. Cancer Med.

[5] Xiao H., Zhang Y., Kong D., Li S., Yang N. (2020). The Effects of Social Support on Sleep Quality of Medical Staff Treating Patients with Coronavirus Disease 2019 (COVID-19) in January and February 2020 in China. Med Sci Monit.

[6] Hendin A., La Rivière C.G., Williscroft D.M., O’Connor E., Hughes J., Fischer L.M. (2020). End-of-life care in the Emergency Department for the patient imminently dying of a highly transmissible acute respiratory infection (such as COVID-19). CJEM.

[7] Starace F., Ferrara M. (2020). COVID-19 disease Emergency Operational Instructions for Mental Health Departments issued by the Italian Society of Epidemiological Psychiatry. Epidemiol Psychiatric Sci.

[8] Hunter D., Wright C., Pearson S. (2019). Employing positive psychology to improve radiation therapy workplace culture. J Med Radiat Sci.

[9] Haslam S.A., Reicher S. (2006). Stressing the group: Social identity and the unfolding dynamics of responses to stress. J Appl Psychol.

[10] Richins M.T., Barreto M., Karl A., Lawrence N. (2019). Incidental fear reduces empathy for an out-group’s pain. Emotion.

[11] Croucher S.M., Nguyen T., Rahmani D. (2020). Prejudice Toward Asian Americans in the Covid-19 Pandemic: The Effects of Social Media Use in the United States. Front Commun.

[12] Influs M., Masalha S., Zagoory-Shaon O., Feldman R. (2019). Dialogue intervention to youth amidst intractable conflict attenuates stress response to outgroup. Horm Behav.

[13] Dovidio J.F., Gaertner S.L., Gladys K. (2000). Group identity and intergroup relations The common in-group identity model. Adv Group Processes.

[14] Christ O, Kauff M. Intergroup Contact Theory. In: Sassenberg K, Vliek MLW, editors. Social Psychology in Action: Evidence-Based Interventions from Theory to Practice. Springer International Publishing; 2019, p. 145–161. https://doi.org/10.1007/978-3-030-13788-5_10.

[15] Waters L. (2012). Predicting Job Satisfaction: Contributions of Individual Gratitude and Institutionalized Gratitude. Psychology.

[16] Martini M., Gratitude C.D. (2014). or the Positive Side of the Relationship with Patients. Development and First Validation of New Instruments: A Scale of Gratitude Perceived by Operators and a Scale of Support Offered by the Gratitude Expressed by Their Patients. Psychology.

[17] Ghandeharioun A., Azaria A., Taylor S., Picard R.V. (2016). “Kind and Grateful”: A Context-Sensitive Smartphone App Utilizing Inspirational Content to Promote Gratitude. Psychol Well-Being.

[18] Semi di Gratitudine al Gemelli ART. http://books.kbms.it/books/vcms 2021 [accessed 06 April 2021].

